# Erosion potential of the Yangtze Delta under sediment starvation and climate change

**DOI:** 10.1038/s41598-017-10958-y

**Published:** 2017-09-05

**Authors:** H. F. Yang, S. L. Yang, K. H. Xu, H. Wu, B. W. Shi, Q. Zhu, W. X. Zhang, Z. Yang

**Affiliations:** 1 State Key Laboratory of Estuarine and Coastal Research, East China Normal University, Shanghai, 200062 China; 2 Department of Oceanography and Coastal Sciences, Louisiana State University, Baton Rouge, LA 70803 USA; 3 Coastal Studies Institute, Louisiana State University, Baton Rouge, LA 70803 USA; 4 Ministry of Education Key Laboratory for Coast and Island Development, Nanjing University, Nanjing, 210093 China; 5 Department of Hydraulic Engineering, Faculty Civil Engineering and Geosciences, Delft University of Technology, P.O. Box 5048, 2600 GA Delft, The Netherlands; 6 College of Marine Geosciences, Ocean University of China, 238 Songling Rd., Qingdao, 266100 China

## Abstract

Deltas are widely threatened by sediment starvation and climate change. Erosion potential is an important indicator of delta vulnerability. Here, we investigate the erosion potential of the Yangtze Delta. We found that over the past half century the Yangtze’s sediment discharge has decreased by 80% due to the construction of >50,000 dams and soil conservation, whereas the wind speed and wave height in the delta region have increased by 5–7%, and the sea level has risen at a rate of 3 mm/yr. According to hydrodynamic measurements and analyses of seabed sediments, the period when bed shear stress due to combined current-wave action under normal weather conditions exceeds the critical bed shear stress for erosion (*τ*_*cr*_) accounts for 63% of the total observed period on average and can reach 100% during peak storms. This explains why net erosion has occurred in some areas of the subaqueous delta. We also found that the increase with depth of *τ*_*cr*_ is very gradual in the uppermost several metres of the depositional sequence. We therefore expect that the Yangtze subaqueous delta will experience continuous erosion under sediment starvation and climate change in the next decades of this century or even a few centuries.

## Introduction

Deltas, which are depositional systems of riverine sediments, are densely populated socio-economic centres. However, many deltas are now threatened by human-induced sediment starvation and climate change^1–3^. In extreme examples, such as the Nile and Colorado deltas, sediment discharges have decreased to almost zero. In the Mississippi, Yellow and many other rivers, the sediment discharges to the sea have also declined by 60–90% in recent decades^4–6^. This sediment starvation in deltas has mainly been caused by river damming, although water diversion and soil conservation within watersheds also play roles^7–9^. Additionally, global warming has been an important scientific and environmental issue^10, 11^. Under these conditions, some studies have revealed that sea surface wind speeds and wind wave heights have increased^12, 13^. Deltas’ response to sediment starvation and hydrodynamic increase depend on the contrast between the marine hydrodynamics and seabed sediment properties and can differ between rivers. Previous studies on delta erosion have mainly focused on coastline retreat^6, 14–18^. Less is known about the response of broad subaqueous deltas to sediment starvation due to a lack of bathymetric and stratigraphic data. Detecting erosion or determining the erosion potential in delta coasts is very important to both ecology and management because many oil pipelines and optic cables are shallowly buried beneath the seabed of deltaic coasts. Thus, an urgent need exists to investigate the erodibility and predict the erosion potential of subaqueous deltas, particularly for large rivers.

**Figure 1:**
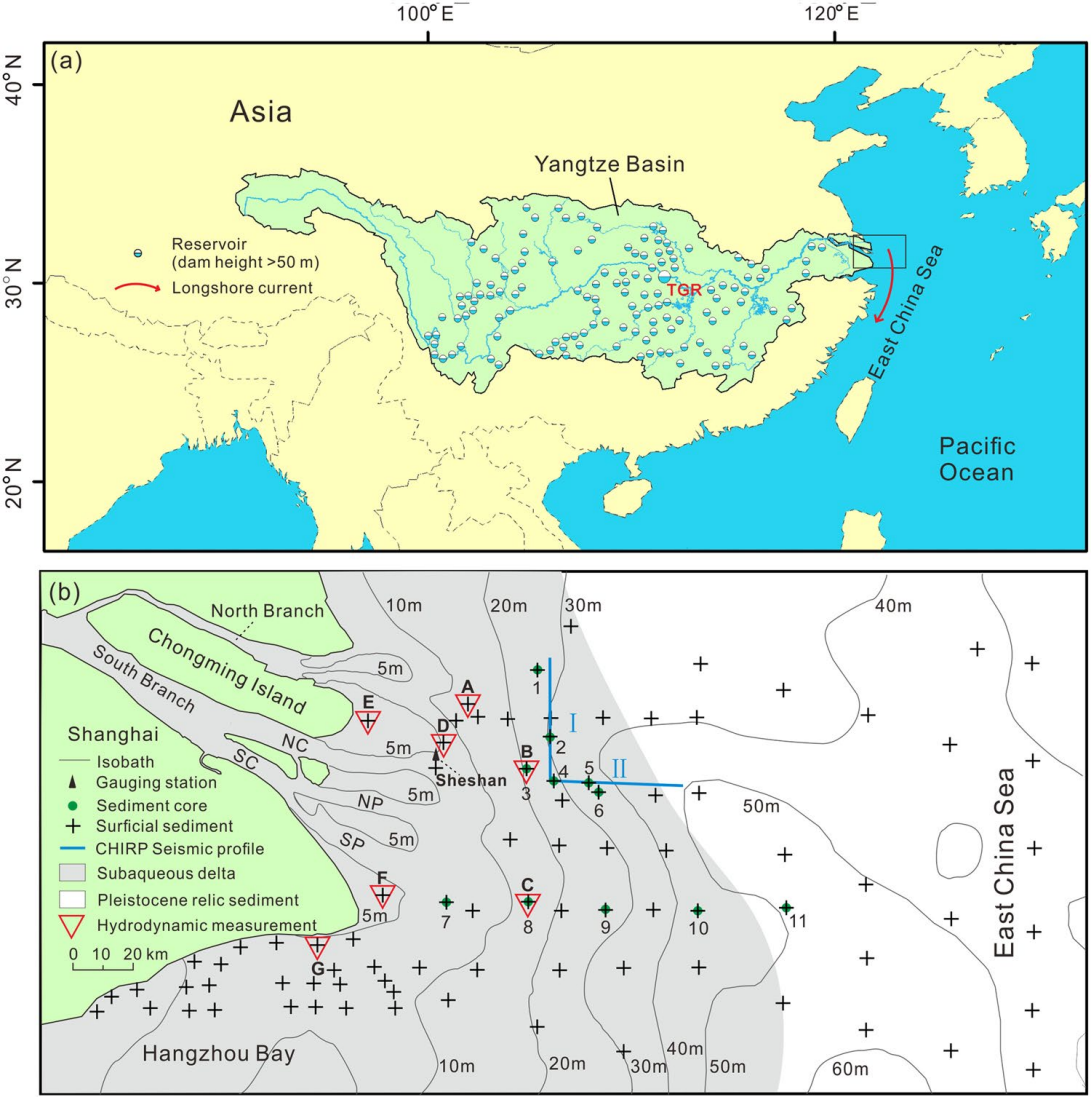
Location and background of the Yangtze River Delta (**a**) and the sediment sampling and hydrodynamic observation sites (**b**). Main reservoirs with dam height >50 m in the Yangtze basin are shown in Fig. 1a. In Fig. 1b, the area in grey represents the subaqueous delta, and the area in white represents the Pleistocene relic sand. NC: North Channel, SC: South Channel, NP: North Passage, and SP: South Passage. The figures were created using ArcGIS 10.1 (www.esri.com/software/arcgis) and CorelDRAW Graphics Suite X6 (http://www.coreldraw.com/en/product/graphic-design-software).

The Yangtze River is one of the world’s largest rivers in terms of water discharge (900 km^3^/yr) and sediment load (500 Mt/yr before decline; Mt: million tons) and is the largest in terms of its watershed population (450 million)^6, 19^. In recent decades, approximately 50,000 dams have been constructed within the Yangtze watersheds. Among them, the world’s largest hydropower project, the Three Gorges Reservoir (TGR)^20^, began its operation on the main stem of the Yangtze River in 2003, and a series of cascade dams, whose cumulative reservoir capacity exceeds that of the TGR (Fig. 1a), began their operation in the largest and previously undammed Jinshajiang sub-basin in 2012–2015. In addition, large-scale soil conservation has been conducted^9^. Thus, the Yangtze’s sediment discharge to the sea has decreased by nearly 80% (<120 Mt/yr in 2013–2015). Yang *et al*.^21^ reported erosion in an 1800-km^2^ area of this delta between 2004 and 2007. However, a later study found that the accumulation in this area had rebounded^22^. Recently, new evidences of erosion in the Yangtze subaqueous delta have been found, either within areas that were not previously studied or due to updated data for the previously studied areas^23–25^. The above controversy reflects the complexity of a delta’s response to sediment starvation. The entire area of the Yangtze subaqueous delta exceeds 10,000 km^2^. For most of this area, bathymetric data in high spatial and temporal resolutions are unavailable for ascertaining the morphological response to sediment starvation. Thus, we must develop a hydrodynamic-sedimentary approach to examine the morphological trends of the broad delta.

During our study, the interannual variations of tidal ranges were relatively small with the difference between the minimum and maximum <6%^25^. Our observations were mainly conducted in July and August under normal weather conditions, when tidal ranges were close to mean annual value with the difference <5%^26^. Compared to the monthly and interannual variations of tidal ranges, the difference between spring and neap tides are quite large^27^. Therefore, observations lasting for more than 2 tidal cycles during both spring and neap tides were conducted to study the average condition of hydrodynamics in the Yangtze Delta. In this study, we aim to investigate the hydrodynamic mechanisms of delta erosion under sediment starvation and predict the future erosion potential of the Yangtze subaqueous delta. Our objectives are to (1) determine the bed shear stress due to the combined current-wave action (*τ*_cw_), (2) determine the critical shear stress for the erosion of sediment (*τ*_cr_), (3) compare the values of *τ*_cw_ with those of *τ*_cr_ and quantify the sediment erodibility, (4) ascertain the thickness of sediment that is erodible under sediment starvation and combined current-wave action, (5) discuss whether this subaqueous delta will experience extensive erosion and how long this erosion could continue.

## Results

### Temporal variations in environmental drivers

**Figure 2:**
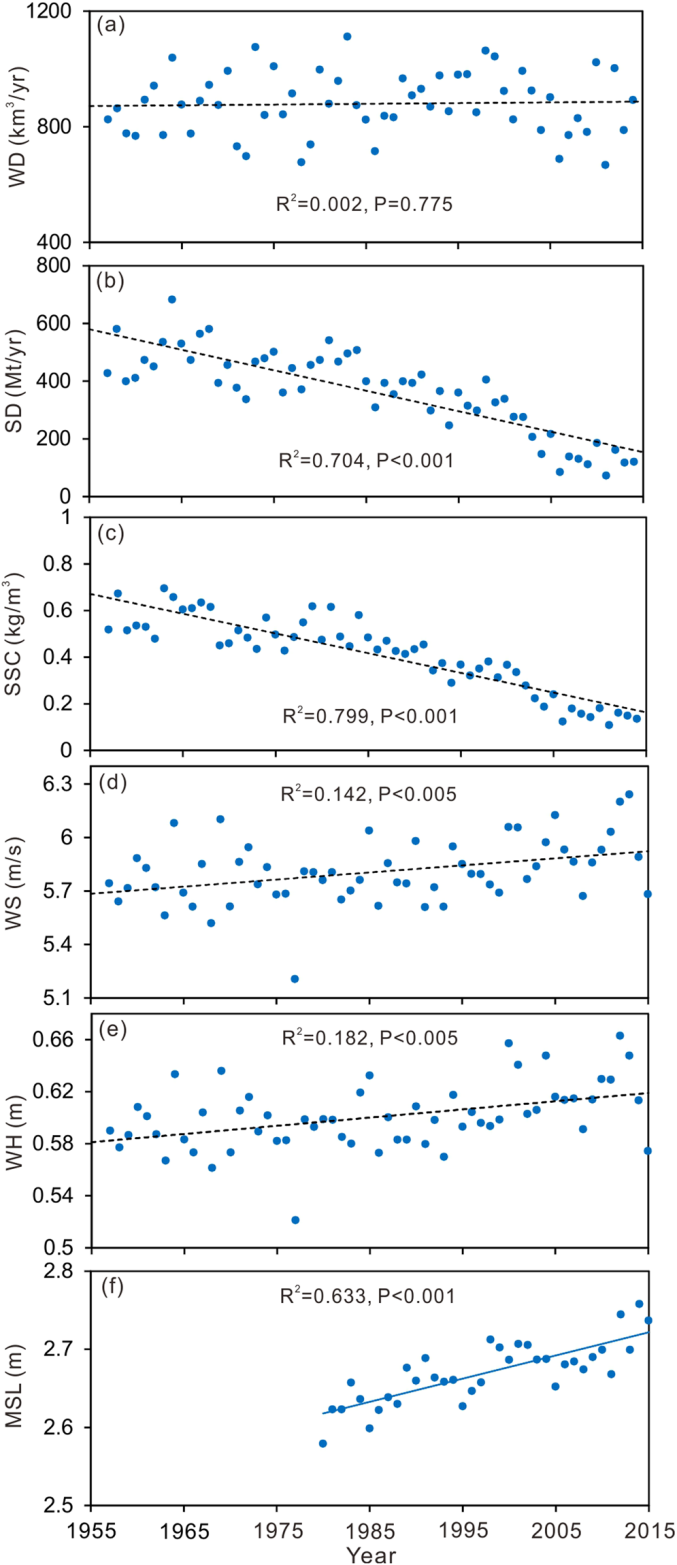
Temporal changes of the annual water discharge (**a**), sediment discharges (**b**) and suspended sediment concentration (**c**) in the Yangtze River, annual average wind speed (**d**) and significant wave height (**e**) at Sheshan station, and annual mean sea level (above the Astronomic Lowest Tide) in the Northern East China Sea (**f**). The location of Sheshan station was noted in Fig. 1b. WD: water discharge, SD: sediment discharge, SSC: suspended sediment concentration, WS: wind speed, WH: wave height and MSL: mean sea level.

No evident trend (P ≫ 0.050) in annual water discharge from the Yangtze River was found, which has remained at a steady level of approximately 900 km^3^/yr since the mid 1950s (Fig. 2a). However, the riverine sediment discharge decreased very significantly (P < 0.001) from approximately 500 Mt/yr in the 1950s to only approximately 130 Mt/yr in the 2010s (Fig. 2b). Thus, the riverine suspended sediment concentration exhibited a temporal trend (P < 0.001) similar to the riverine sediment discharge (Fig. 2c). In the meantime, slight increasing trends in both the annual wind speed (P < 0.005) and significant wave height (P < 0.005) were found during the past approximately 60 years (Fig. 2d,e). The annual average wind speed increased from 5.70 m/s in the 1950s to 6.00 m/s in the 2010s, and the annual average significant wave height increased from 0.58 m in the 1950s to 0.63 m in the 2010s. The mean sea level in the northern East China Sea showed a significant rising trend (P < 0.001), and the average rate of sea level rise was approximately 3 mm/yr during the past approximately 35 years (Fig. 2f).

### Variability of *τ*_*cw*_

**Figure 3:**
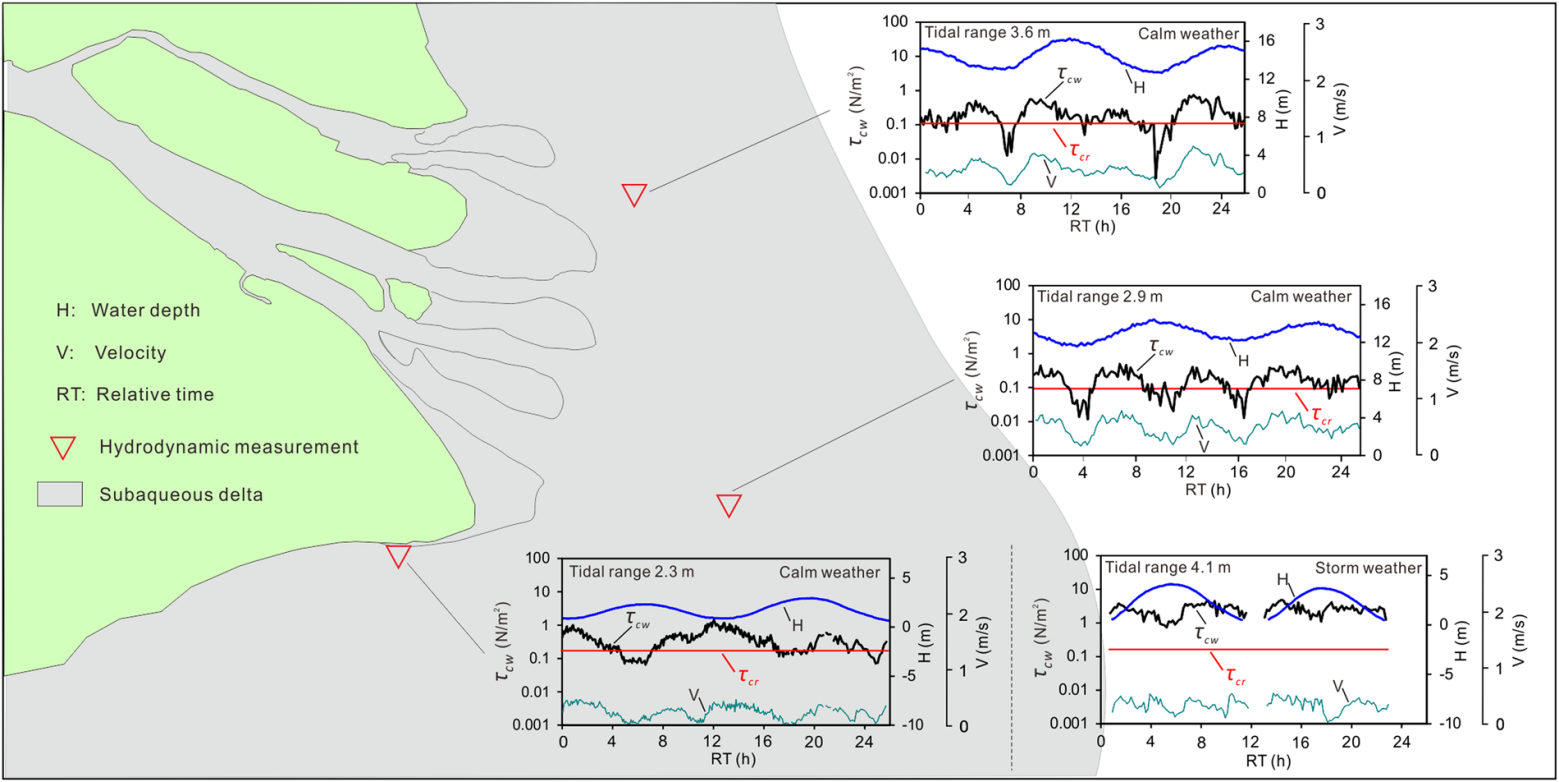
Four examples showing intratidal variations in the water depth, near-bed velocity and combined current-wave shear stress (*τ*_*cw*_) compared to the critical bed shear stress (*τ*_*cr*_) at the observation stations. A complete exhibition of all the observations was in the attached Supplementary Fig. S1. The figures were created using ArcGIS 10.1 (www.esri.com/software/arcgis) and CorelDRAW Graphics Suite X6 (http://www.coreldraw.com/en/product/graphic-design-software).

*τ*_*cw*_ was highly variable in time and space. Under normal wind conditions, the *τ*_cw_ values ranged from 0.08 to 0.67 N/m^2^ and were 0.29 N/m^2^ on average. The lowest *τ*_*cw*_ values were usually found in slack waters at low and high tides, and the peak *τ*_*cw*_ values tended to occur at mean tidal level, when the current velocity was the greatest. The *τ*_*cw*_ during spring tides was 2.1 times greater than that during neap tides on average. The time-averaged *τ*_*cw*_ values at A, B, C, D, E, F and G were 0.16, 0.28, 0.25, 0.39, 0.18, 0.28 and 0.48 N/m^2^, respectively (Table 1). During a peak storm event (spring tide), the *τ*_*cw*_ values at a shallow site (G) ranged from 0.73 to 4.79 N/m^2^ and were 2.56 N/m^2^ on average, which was 4.5 times greater than that under normal wind conditions (spring tide) (Fig. 3, Supplementary Fig. S1 and Table 1).

**Table 1:** Tide-averaged *τ*_*cw*_ and *τ*_*cr*_ and percent of period when *τ*_*cw*_ > *τ*_*cr*_ in observations at the seven sites.

			Site A	Site B	Site C	Site D	Site E	Site F	Site G
*τ*_*cw*_ (N/m^2^)	Normal winds	Neap tide	0.08	0.23	0.18	0.11	0.16	0.15	0.38
		Spring tide	0.24	0.32	0.31	0.67	0.20	0.41	0.57
		Average	0.16	0.28	0.25	0.39	0.18	0.28	0.48
	Peak storm	Spring tide	ND	ND	ND	ND	ND	ND	2.56
*τ*_*cr*_ (N/m^2^)	Surficial sediment		0.11	0.09	0.09	0.14	0.18	0.23	0.16
	Core sediment (average)		ND	0.10	0.09	ND	ND	ND	ND
Period of *τ*_*cw*_ > *τ*_*cr*_ in observation (%)	Normal winds	Neap tide	31	66	73	23	41	21	79
		Spring tide	82	81	91	78	58	78	81
		Average	57	74	82	51	50	50	80
	Peak storm	Spring tide	ND	ND	ND	ND	ND	ND	100

ND: No data are available. Each measurement was continuously conducted for 26 hours.

The *τ*_*cw*_ in the Yangtze’s subaqueous delta was generally dominated by tidal action. The wave-induced shear stress at the depth of 20 m decreased by >98% compared to that at the depth of 2 m. More than 95% of the *τ*_*cw*_ at the observation sites with water depths that exceeded 10 m could be attributed to tidal action. However, the *τ*_*cw*_ on the intertidal flats was dominated by wave action during storm events and very shallow water phases of tidal cycles (water depth < 0.5 m) under normal weather conditions. The mean wind speed at site G increased from 6.3 m/s under normal weather conditions to 11.5 m/s during peak storms (Table 2), and in response, the mean significant wave height increased from 0.22 m to 0.65 m. The mean *τ*_*w*_ was only 0.05 N/m^2^ under normal weather conditions, while it increased to 0.85 N/m^2^ during peak storms (Supplementary Fig. S2). The maximum value for wave-induced shear stress even reached 1.62 N/m^2^, which represented an increase of more than one order of magnitude compared to that under normal weather conditions. Meanwhile, the striking changes were also detected during pre-peak storm and post-peak storm periods compared to that under normal weather conditions at site G. The mean wind speeds were 9.0 m/s during pre-storm period and 7.8 m/s during post-peak storm period, and the mean significant wave heights were 0.29 m and 0.30 m accordingly. Therefore, the mean *τ*_*w*_ were 0.23 N/m^2^ during pre-peak storm period and 0.24 N/m^2^ during post-peak storm period.

**Table 2:** Descriptions of the *in situ* observations at the seven sites.

	Site A	Site B	Site C	Site D	Site E	Site F	Site G
Mean water depth (m)	14	21	13	7.5	0.72	1.7	2.2
Mean wind speed (m/s)*	6.2	6.0	5.9	4.4	5.9	4.9	6.3/11.5
Wind strength	Normal	Normal	Normal	Normal	Normal	Normal	Normal/stormy
Tide type	Neap, Spring	Neap, Spring	Neap, Spring	Neap, Spring	Neap, Spring	Neap, Spring	Neap, Spring
Landform background	Delta front off the NB	Delta front off the NC	Delta front off the NC	Subtidal slope between NB and NC	Tidal flat between NB and NC	Tidal flat between SP and HB	Tidal flat in north HB

A, B, C, D, E, F and G represent the comprehensive observation sites in Fig. 1b. NB: North Branch. NC: North Channel. NP: North Passage. SP: South Passage. HB: Hangzhou Bay. *Based on hourly wind speed series from the European Centre for Medium-Range Weather Forecasts (ECMWF) (http://www.ecmwf.int/). The area behind the oblique line indicates a storm event.

Based on the historical data at Sheshan station, the hourly average of wind speed in the Yangtze Delta ranged from 0.01 to 24.1 m/s during the past 60 years, and the multi-year average was 5.8 m/s. Meanwhile, the hourly average of significant wave height ranged from 0.01 to 3.1 m, and the multi-year average was 0.6 m. During our observation of peak storms, the mean wind speed was 11.5 m/s and the mean significant wave height was 1.4 m at Sheshan station, which were both higher than 98% of the records (Supplementary Fig. S3). Considering that storm events (wind speed > 10.8 m/s, grade of storms: http://www.sac.gov.cn/) accounted for 4% of the whole time (Supplementary Fig. S3a), our observation of peak storms was just at a moderate level among all the storms. During the most powerful storms of the past 60 years, the *τ*_*w*_ on the intertidal flats could be more than the double of our measured values in this observation.

### Spatial distributions of sediment grain size, water content and *τ*_*cr*_

**Figure 4:**
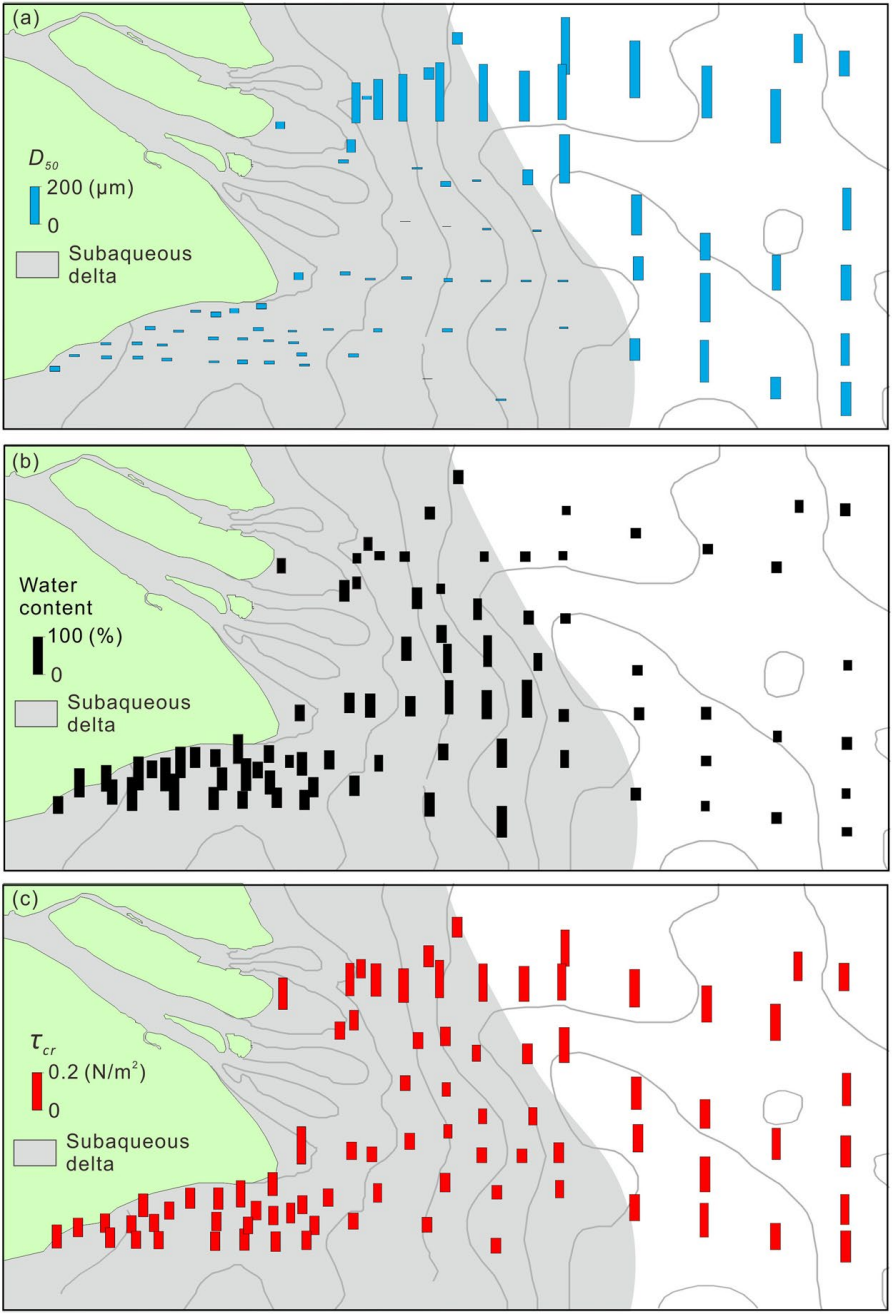
Histograms of the median size (*D*_50_, in blue) (**a**), water content (in black) (**b**) and the *τ*_*cr*_ of the surficial sediments (in red) (**c**). The figures were created using ArcGIS 10.1 (www.esri.com/software/arcgis) and CorelDRAW Graphics Suite X6 (http://www.coreldraw.com/en/product/graphic-design-software).

The surficial sediment of the Yangtze Delta is overall small in grain size and high in water content. The median size (*D*_50_) of the surficial sediments in the study area ranged from 5 to 294 μm and was 82 μm on average. In the Yangtze subaqueous delta, the *D*_50_ values averaged 37 μm, which is significantly lower than the average *D*_50_ of the Pleistocene relic sediments (Fig. 1b) in the deeper East China Sea (198 μm). Differences in *D*_50_ were also found within the Yangtze subaqueous delta. The *D*_50_ values averaged 244 μm in the delta front off the North Branch and averaged only 18 μm in the areas offshore of the South Branch’s outlets (Fig. 4a). The water content of the surficial sediments ranged from 25% to 106% and was 46% on average. The water content in the Pleistocene relic sands averaged 30%, whereas the water content in the Yangtze subaqueous delta averaged 53%. The spatial pattern of the water content was opposite to that of the grain size (Fig. 4b).

The *τ*_*cr*_ values of the delta’s surficial sediments ranged from 0.08 to 0.23 N/m^2^ with an average of 0.13 N/m^2^, which was overall slightly higher than that in the Yangtze subaqueous delta (0.11 N/m^2^) and much lower than that of the Pleistocene relic sands (0.18 N/m^2^) (Fig. 4c). The period when *τ*_*cw*_ > *τ*_*cr*_ comprised 21–79% (48 ± 24%) of the observations during neap tides and 58–91% (78 ± 10%) of the observations during spring tides under normal winds at the observation sites. At a shallow site, *τ*_*cw*_ was always significantly larger than *τ*_*cr*_ during peak storm events, and the period when *τ*_*cw*_ > *τ*_*cr*_ comprised 100% of the observations (Table 1 and Fig. 3).

**Figure 5:**
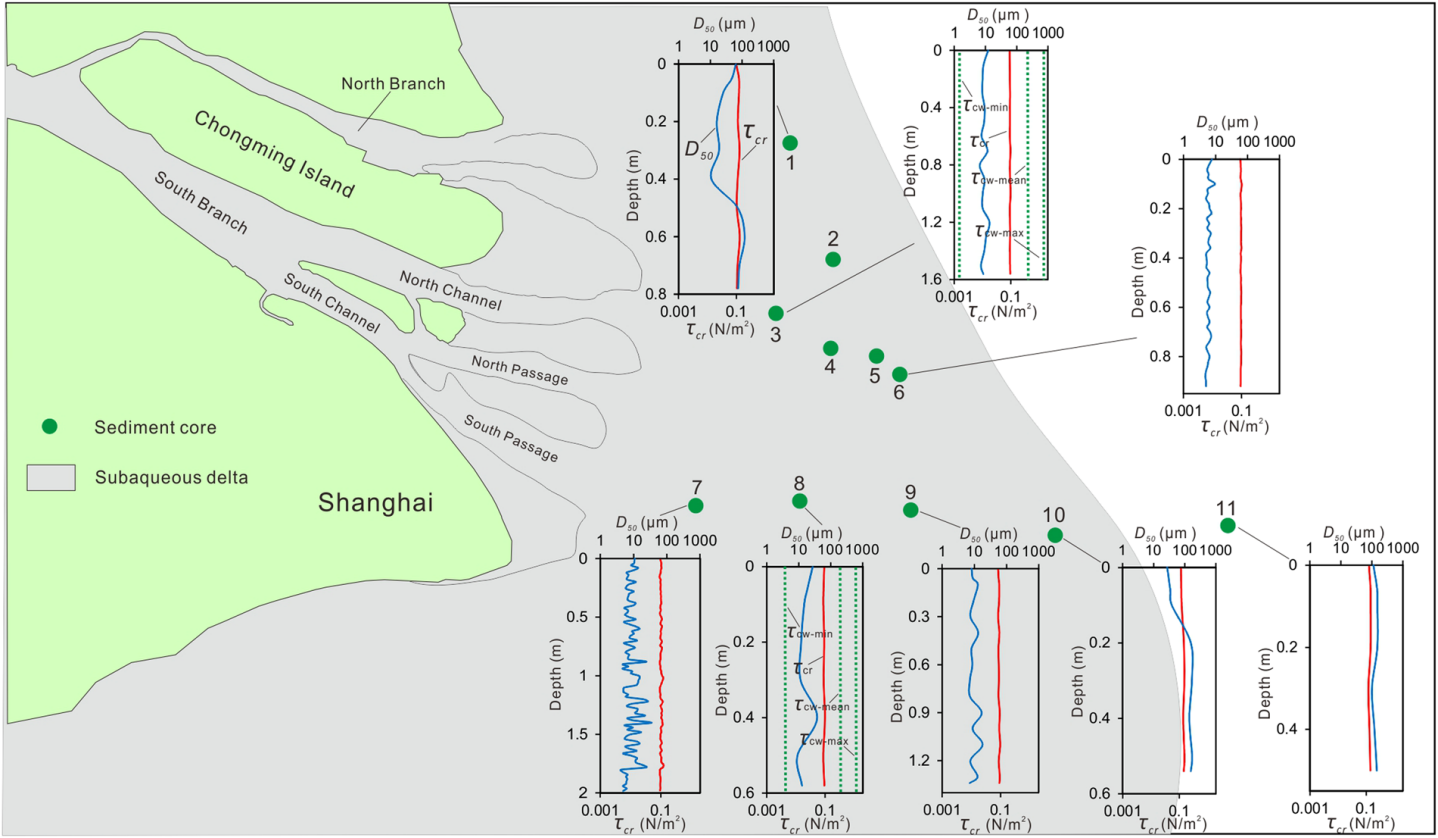
Vertical changes of the *D*_50_ (in blue) and *τ*_*cr*_ of the sediment cores (in red), and the measured characteristic *τ*_*cw*_ values (in green) compared to *τ*_*cr*_ values at the observation sites (*τ*_*cw*-min_, *τ*_*cw*-mean_ and *τ*_*cw*-max_ represent the minimal, mean and maximum *τ*_cw_ values, respectively). The figures were created using ArcGIS 10.1 (www.esri.com/software/arcgis) and CorelDRAW Graphics Suite X6 (http://www.coreldraw.com/en/product/graphic-design-software).

**Figure 6:**
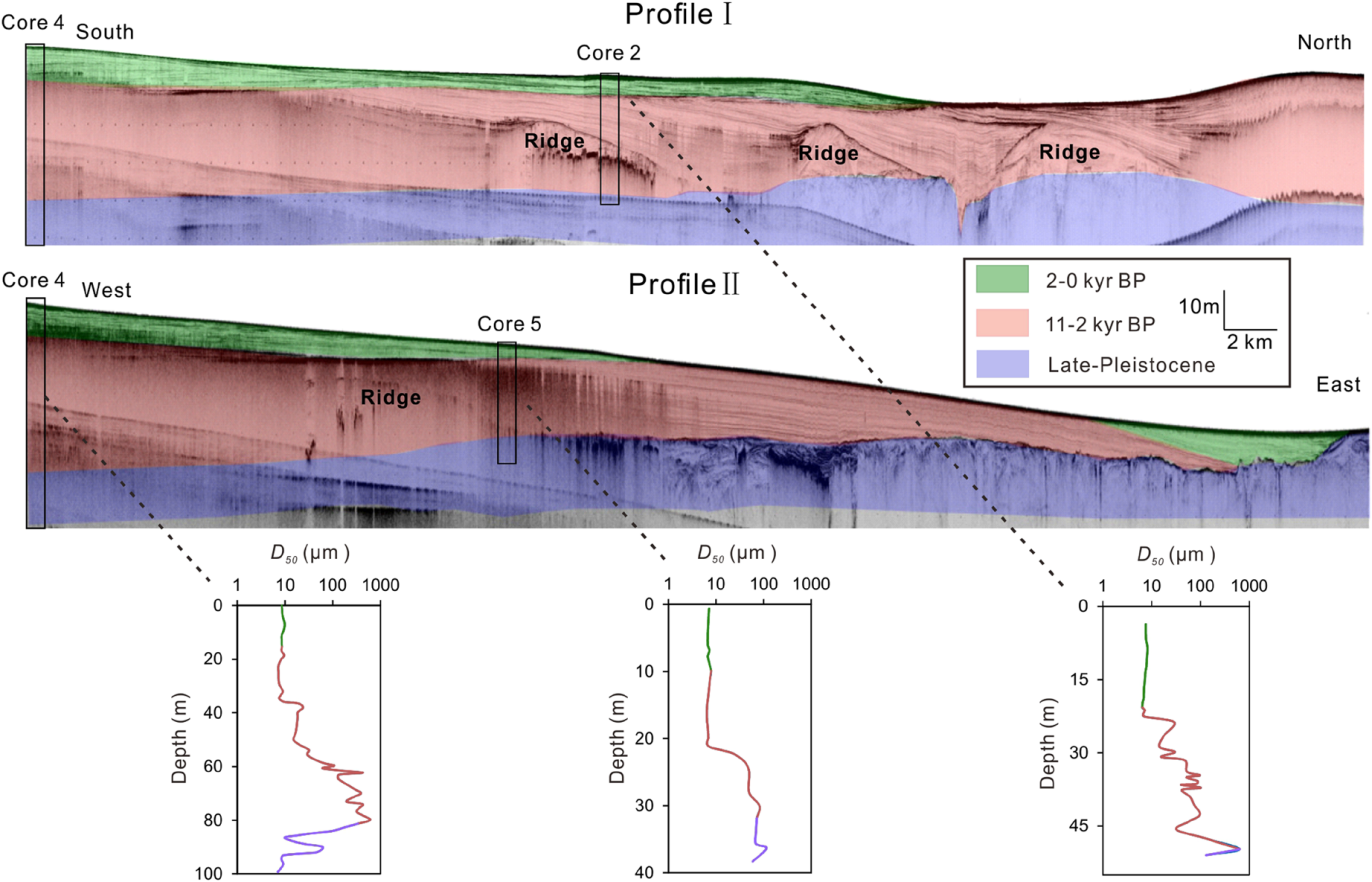
Two CHIRP seismic profiles and three cores in the Yangtze delta (modified from *Xu et al*.^57^). Three units are coloured by green, red and blue from top to bottom. The vertical variations in core sediment grain size are also shown in different colours. BP = Before Present, where present is 1950 A.D.

The vertical trend in the *τ*_*cr*_ of the sediment cores in the upper most several metres was generally non-significant, although a slight trend of increase with depth was found in a few cores (Fig. 5). Based on the hydrodynamic measurements, the *τ*_*cr*_ for the upper most several metres was much higher than *τ*_*cw-min*_ and much lower than *τ*_*cw-mean*_ and *τ*_*cw-max*_ (Fig. 5). Based on CHIRP subbottom seismic profiling measurements, the sediments deposited during the past 2000 years is approximately 10–20 m in thickness (Fig. 6). These modern sediments are fine grained and have a similar median size to the surficial sediments.

## Discussion

A comparison between the *τ*_*cw*_ and *τ*_*cr*_ values at the observation sites suggests that the Yangtze subaqueous delta was widely erodible during most of the study period. The seabed at the observation sites with water depths from <1 m to 21 m (Fig. 1b and Table 2) was not erodible only during slack waters under calm weather, which generally comprised less than 40% of the study period (Table 1). It can be inferred that the erodibility in the subaqueous delta deeper than 21 m is comparable to the erodibility at the observation sites due to the similarity of tidal conditions^28^ and comparable *τ*_*cr*_ values (Fig. 4c). The lack of a significant vertical trend in the *τ*_*cr*_ values of the core sediments suggests that the deposit layers are relatively homogenous for the upmost approximately 20 metres. This vertical stability does not reflect the seasonal changes in the erodibility of the real-time surficial sediment. According to the present Yangtze’s sediment discharge (ca. 120 Mt/yr), its seasonal variability, and the subaqueous delta area (>10, 000 km^2^), the deposition in the subaqueous delta during summer can be several centimetres in thickness. The erodibility of this fresh deposition must be even lower than that shown above (the upper 30 cm of the sediment sampled during the dry season). This low erodibility was also verified using known erosion dynamics produced by other researchers. For example, vertical degradation appeared in the mouth area of the North Branch (Fig. 1b) between 1997 and 2012 with a mean rate of −6.7 cm/yr and margin recession was also noted in the subaqueous delta front offshore of the South Branch^25^. Du *et al*.^24^ also reported an erosion rate of approximately 2 cm/yr at the mouth area off the South Branch. The amount of sediment eroded from the subaqueous delta was more than 1 billion tons from 2002 to 2013 and mainly occurred between −6.4 m and −19 m isobaths^29^. In contrast, high and dense vegetation in the salt marshes of the Yangtze Delta efficiently attenuates waves and currents^30, 31^, and the *τ*_*cw*_ is generally lower than the *τ*_*cr*_ of the bed sediments^32^, which suggest a much lower erodibility. However, marsh edge collapses can result in the loss of salt marshes^33, 34^. Tidal flat erosion is expected to create steep slope along the lower marsh edge, and marsh edge collapses subsequently shrinks salt marshes.

An erodible seabed does not necessarily lead to net erosion. Both erosion and deposition events occur during a tidal cycle. Net seabed erosion/accretion depends on the balance between the erosion flux and deposition flux^35–37^. The erosion flux is determined by the sediment erodibility, whereas the deposition flux is determined by the suspended sediment concentration^38, 39^. Thus, net erosion of the Yangtze Delta can be expected under sediment starvation, given the erodible seabed. Longshore currents play an important role in residual sediment transport. The suspended sediment concentrations in the Yangtze Estuary and adjacent coastal waters has significantly reduced in response to the decrease in riverine sediment discharge^40, 41^, which would decrease the deposition flux and may result in net erosion through or within the subaqueous delta. In fact, a few of the most recent studies support this finding^24, 25^. Of course, the morphological response to a decline in the riverine sediment discharge may spatially vary, and accretion may continue somewhere in the large subaqueous delta^22^. Over the next several decades, the sediment discharge from the Yangtze River will further decrease as many new dams are constructed in the Yangtze basin^27^, and continued delta erosion can be expected, particularly in the subaqueous delta front, where longshore currents develop. The CHIRP subbottom seismic profiles and vertical distribution of grain size (Fig. 6) suggest that *τ*_*cr*_ is low and the strata are most likely erodible for the uppermost 10-20 m of the subaqueous delta deposit. The erosion rate of the Yangtze subaqueous delta was 3–7 cm/yr over the past one to two decades^21, 24, 25^. Assuming that the delta experiences a similar erosion rate under decreased river sediment supply, increased coastal hydrodynamics and a slight downward increase in *τ*_*cr*_, the erosion of the uppermost 10–20 m sediments will likely continue for the next decades of this century or even a few centuries.

Presumably, the erodibility and morphological trends of the world’s deltas under river damming can differ greatly because the major influencing factors, namely the delta sediment properties, the local hydrodynamics for sediment resuspension, and the presence of longshore currents for the residual transport of sediment, can greatly change. The Yangtze Delta can likely be described as having a low *τ*_*cr*_, high *τ*_*cw*_ and relatively strong longshore currents. Prior to river damming, the Yangtze Delta experienced rapid accretion due to the abundant fine-grained sediment supply^21, 42^. In other words, the uppermost several metres of sediment layers were not very compacted. The Yangtze Delta is under the impact of meso-macro tides and is exposed to the sizeable East China Sea, which suggests a high *τ*_*cw*_. The monsoon-driven longshore currents off the Yangtze River mouth are relatively strong, with residual flow velocities that reach approximately 30 cm/s^43^. In comparison, the Mississippi and Nile deltas are under the impact of micro tides^44, 45^. However, perhaps many of the deltas would experience erosion under dam-generated sediment starvation, although deltas differ in terms of their erodibility. For example, the Mississippi subaqueous delta experienced severe erosion during storms^46^. Long-term coastal recession that was associated with river damming and wave-induced longshore currents was found in the Nile delta^6, 47^.

Last but not least, increasing trends of longshore currents and wind waves are expected because global warming and sea surface wind speed increase have been extensively reported^10, 11, 13^ and because of the results presented in Fig. 2. For example, *Gulev and Grigorieva* ^12^ reported positive trends of significant wave height over the North Pacific with a maximum of 8–10 cm/decade in the northeast Pacific. In addition, increasing water depth due to sea level rise^48, 49^ can also result in increased wave height in the intertidal zone^50^.

## Conclusions

The upper several metres of fine-grained deposit in the Yangtze’s subaqueous delta is overall high in water content, which leads to low *τ*_*cr*_ across wide areas. This delta is under the influence of meso to macro tides and exposed to the open sea, resulting in a high *τ*_*cw*_. The seabed of this delta tends to be erodible during peak tidal flow phases but non-erodible during slack water phases. Under fair weather conditions, the erodible period comprises nearly 50% of the duration during neap tides and nearly 80% of the duration during spring tides. During storm events, the shallow seabed can always be erodible. Dam-induced sediment starvation reduces the deposition flux during non-erodible periods. Thus, the erosion flux may exceed the deposition flux within the tidal cycle. Net delta erosion can be seen together with strong longshore currents, which can transport sediment southward. Over the next several decades, continuous erosion can be expected for the uppermost several metres of sediments in the subaqueous delta, considering that the Yangtze sediment discharge will further decrease due to the construction of new dams and that the energy of tides and waves in coastal ocean will likely continue to increase under global warming and sea level rise. Delta erosion will threaten ecosystems and engineering facilities (e.g., shallowly buried oil, gas pipelines, optical cables and seawalls). Subaqueous deltaic erosion and the processes controlling sediment erodibility need to be one of the key topics of studies on global coastal protection and restoration.

## Study area

### The Yangtze River and its delta

The Yangtze River originates on the Qinghai–Tibet Plateau and flows 6,300 km eastward to the East China Sea (Fig. 1a). Approximately 6,000 years BP, when the post-glacial sea level was near its maximum height, the mouth of the Yangtze River was approximately 300 km inland relative to the present coastline. Since then, the delta advanced due to the deposition of riverine sediments^51^. Over the past 2,000 years, the delta’s progradation has accelerated due to catchment deforestation^26, 52^. However, coastal progradation has significantly slowed during the most recent decades because of the dam-generated decline in the Yangtze’s sediment discharge^53, 54^.

The current Yangtze mouth includes four outlets (Fig. 1b). Prior to the 18th century, the North Branch was the main outlet of the Yangtze’s water and sediment discharge. During the 18th century, however, the major river flow shifted to the South Branch^52^. Now, more than 95% of the Yangtze’s water and sediment flows into the sea via the three outlets of the South Branch^55^. The fine sediments from the Yangtze River formed a modern subaqueous delta off the Yangtze Estuary. Further seaward, this area is covered by Pleistocene relic sands^25^, which were probably winnowed by the currents as the sea level rose since the Last Glacial Maximum. Coarse sands due to severe erosion between 1997 and 2012 were also found in the mouth area of the North Branch^25^ (Fig. 1b). The Yangtze Estuary and adjacent waters are meso-macro tidal and influenced by monsoons. The wind speeds are highly variable, with multi-year averages of 4–5 m/s and a maximum of 36 m/s^32^. During winter, a strong southward longshore current system develops under the influence of northerly monsoon winds (Fig. 1a), transporting a bulk of sediment away from the Yangtze Delta. The deposition of this sediment has formed a Holocene mud wedge that is several hundred kilometres long in the inner continental shelf of the East China Sea^56, 57^.

## Methods

### Datasets

The Yangtze River’s water and sediment discharges were obtained from the Yangtze Water Resources Committee (YWRC), Ministry of Water Resource of China. The data on wind speed and significant wave height at Sheshan Station (Fig. 1b) were downloaded from the European Centre for Medium-Range Weather Forecasts (ECMWF) (http://www.ecmwf.int/). The global sea level data were downloaded from the sea level group of the Commonwealth Scientific and Industrial Research Organization (CSIRO), Australia’s national science agency (http://www.cmar.csiro.au/); these data was updated from *Church and White* ^48^. The mean coastal sea level data for China were derived from the State Oceanic Administration, People’s Republic of China (http://www.soa.gov.cn/).

The top sediment (the uppermost 30 cm) and core sediments were sampled in March 2012 and January–March 2013 using a box sampler and a gravity corer, respectively. Two high-resolution seismic profiles were collected after 2003 using a CHIRP sonar sub-bottom profiler (EdgeTech 0512i), and more details can be found in *Xu et al*.^57^ (Fig. 6). One 600-kHz Acoustic Doppler Current Profiler (ADCP, four flow sensors with a 0.5 m bin size, 0.25 m blanking distance, Teledyne RD Instruments, Inc., California, USA) was used to measure the current velocity profile at sites A, B, C and D (Fig. 1b; Table 2). Another ADCP (1.0 MHz high-resolution profiler, Nortek AS, Norway), placed at 70 cm above the sediment surface and looking downward, was utilized to measure the current velocity profiles on the tidal flats at sites E, F and G (Fig. 1b; Table 2). The blanking distance was 40 cm, and the cell size was set to 2.5 cm. Each velocity profile was collected at a frequency of 1 Hz over a duration of 60 seconds during a burst interval of 5 minutes. The current velocity profile at an interval of 10 seconds continually lasted for at least 26 hours (more than 2 tidal cycles) at each site during both spring and neap tides. An SBE-26plus Seagauge (Sea-Bird Electronics, Washington, USA) was utilized to measure wave heights, wave periods, and water depths, etc., using a self-logging sensor. The sampling rate was set to 4 Hz over a duration of 256 seconds during each burst of 10 minutes. The sediment grain size was analysed in the laboratory using a Laser Diffraction Particle Size Analyser, Beckman-Coulter Ls100Q (Beckman Coulter Inc., California, USA). The water content was defined as the ratio of the water weight to the dry sediment weight.

### Calculation of *τ*_*cr*_ and *τ*_*cw*_

In terms of *τ*_*cr*_ (critical bed shear stress for erosion), when sediments on the seabed are mainly non-cohesive deposits, *van Rijn*’s^58^ equation is acceptable in coastal and estuarine areas. *Taki* ^59^ produced another formula that is widely used for cohesive fine sediments. Although *Zhang* ^60^ found that a value of 50 μm for sediments that contain water was the critical value to distinguish cohesive sediments from non-cohesive sediments, a comparison of the two methods that are used to calculate *τ*_*cr*_ was still conducted. When the sediments were finer than 50 μm, the results from *van Rijn* ^58^ were apparently smaller, which did not match practical situations.

When the sediment was finer than 50 μm, *τ*_*cr*_ was calculated as follows^59^:

1 $${\tau }_{cr}=0.05+\beta {\{\frac{1}{{[(\pi /6)(1+{\rm{s}}W)]}^{1/3}-1}\}}^{2}$$

where *β* = 0.3 (for relatively high water content), *w* is the water content, s = (*ρ*_s_/*ρ*_w_)−1, *ρ*_s_ is sediment particle density, and *ρ*_w_ is seawater density.

When the sediment was coarser than 50 μm, *τ*_*cr*_ was calculated as follows^58^:

2 $${\tau }_{cr}={\theta }_{cr}({\rho }_{s}-{\rho }_{w})g{{\rm{d}}}_{{\rm{50}}}$$

in which the critical Shields parameter^61^: *θ*_*cr = *_0.11D_*_^−0.54^, the dimensionless particle size ${\rm{D}}\ast ={[\frac{{\rm{s}}-1{\rm{g}}}{{V}^{2}}]}^{\frac{1}{3}}{{\rm{d}}}_{50}$, s = *ρ*_s_/*ρ*_w_, *θ*_*cr*_ is the dimensionless Shields value, and g is the acceleration of gravity.

*τ*_*c*_ (current shear stress, N/m^2^) was calculated using the following equation^62^:

3 $${\tau }_{c}={\rho }_{w}u{{\ast }_{c}}^{2}$$

where *ρ*_*w*_ is the seawater density (measured to be 1030 kg/m^3^) and *u* * _*c*_ (bed friction velocity, m/s) was derived from linear regression (P < 0.05) based on the logarithmic distribution of vertical current velocity profiles according to the equation between *u*_*c*_(z) and ln z^63^:

4 $${u}_{c}({\rm{z}})=\frac{u{\ast }_{c}}{\kappa \,}\,\mathrm{ln}\,z-\frac{u{\ast }_{c}}{\kappa \,}\,\mathrm{ln}\,{z}_{0}$$

in which z is the height of the velocity profile above the bed (m), z_0_ is the bed roughness length (m), *u*_*c*_(z) is the current speed at z (m/s), and *κ* is Von Karman’s constant (0.4).

*τ*_*w*_ (wave shear stress, N/m^2^) can be calculated as^58^:

5 $${\tau }_{w}=\frac{1}{4}{\rho }_{w}{f}_{w}{{\hat{U}}^{2}}_{\delta }$$

where *f*_*w*_ is the friction coefficient which is determined by the hydraulic regime:

6 $${f}_{w}=\{\begin{array}{ll}2{{\mathrm{Re}}_{w}}^{-0.5}, & {\mathrm{Re}}_{w}\le {10}^{5}\,(\mathrm{laminar})\\ 0.0521{{\mathrm{Re}}_{w}}^{-0.187}, & {\mathrm{Re}}_{w} > {10}^{5}\,(\mathrm{smooth\; turbulent})\\ 0.237{r}^{-0.52}, & (\mathrm{rough\; turbulent})\end{array}$$

in which the wave Reynolds number: ${\mathrm{Re}}_{w}=\frac{{\hat{U}}_{\delta }{\hat{A}}_{\delta }}{\nu }$, the relative roughness: $r=\frac{{\hat{A}}_{\delta }}{{k}_{s}}$, the Nikuradse roughness: *k*_*s*_ = 2.5*D*_50_, *D*_50_ is the median grain size of the bed sediment, and ν is the kinematic viscosity of sea water (m^2^/s).

The peak orbital excursion $({\hat{A}}_{\delta })$ and peak orbital velocity $({\hat{U}}_{\delta })$ can be expressed as:

7 $${\hat{A}}_{\delta }=\frac{H}{2\,\sinh (kh)}$$

8 $${\hat{U}}_{\delta }=\omega {\hat{A}}_{\delta }=\frac{\pi H}{T\,\sinh (kh)}$$

in which H is the wave height (m), the wave number (m^–1^) k = 2π/L, the wave length (m) $L=(g{T}^{{\rm{2}}}/2{\rm{\pi }})\tanh (kh)$, h is the water depth (m), ω is the angular velocity (s^−1^), and T is the wave period (s).

*τ*_*cw*_ (combined current-wave shear stress, N/m^2^) was calculated by ref. 64:

9 $${\tau }_{cw}={\tau }_{c}[1+1.2{(\frac{{\tau }_{w}}{{\tau }_{c}+{\tau }_{w}})}^{3.2}]$$

These models are widely employed to calculate *τ*_*cr*_ and *τ*_*cw*_^32, 39, 65–67^.

## Electronic supplementary material

Supplementary figures
