# Supplementary material for: Erosion potential of the Yangtze Delta under sediment starvation and climate change
Source: Sci Rep. 2017 Sep 5;7:10535. doi: 10.1038/s41598-017-10958-y (PMC5585271; doi:10.1038/s41598-017-10958-y)
Supplement: Supplementary file 1 — Supplementary figures [file 41598_2017_10958_MOESM1_ESM.pdf]

Erosion potential of the Yangtze Delta under sediment starvation and climate change

H.F. Yang, S.L. Yang, K.H. Xu, H. Wu, B.W. Shi, Q. Zhu, W.X. Zhang, Z. Yang

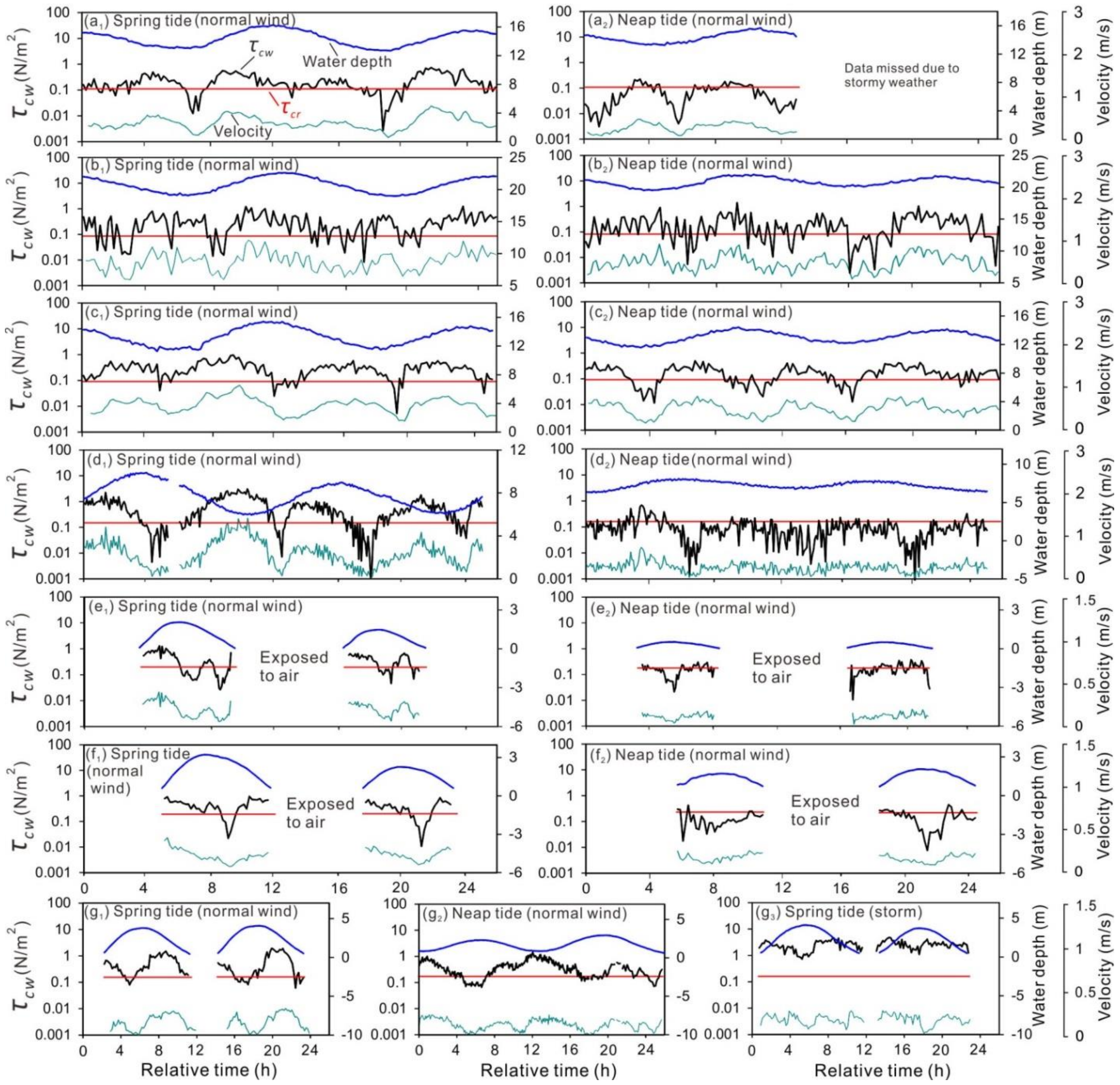

Figure S1. Intratidal variations in the water depth, near-bed velocity and combined current-wave shear stress (τ_{cw}) compared to the critical bed shear stress (τ_{cr}) at the observation stations.

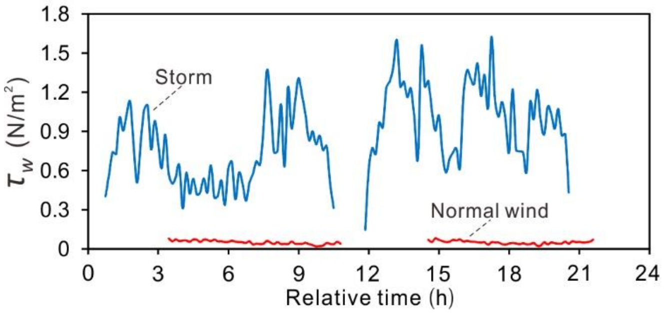

Figure S2. Intratidal variations of wave-induced shear stress (τ_w) under normal weather conditions and during storms at G.

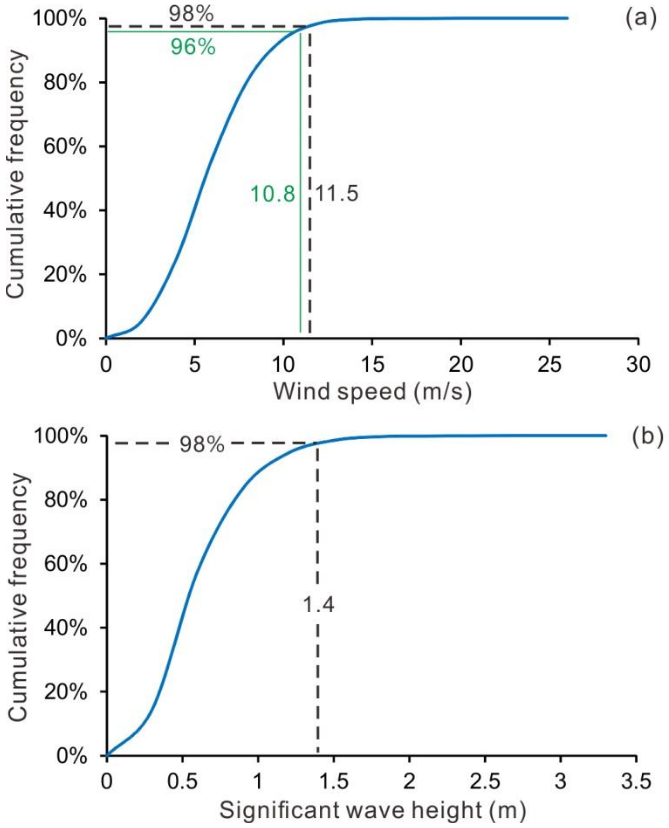

Figure S3. Cumulative frequency curves of wind speeds (a) and significant wave heights (b) during the past 60 years at Sheshan station in the Yangtze Delta.
